# Single-impurity-induced Dicke quantum phase transition in a cavity-Bose-Einstein condensate

**DOI:** 10.1038/s41598-017-07899-x

**Published:** 2017-08-07

**Authors:** Ji-Bing Yuan, Wang-Jun Lu, Ya-Ju Song, Le-Man Kuang

**Affiliations:** 1 Key Laboratory of Low-Dimensional Quantum Structures and Quantum Control of Ministry of Education, Department of Physics and Synergetic Innovation Center for Quantum Effects and Applications, Hunan Normal University, Changsha, 410081 China; 2 College of Physics and Electronic Information Science, Hunan Provincial Key Laboratory of Intelligent Information Processing and Application, Hengyang Normal University, Hengyang, 4210002 China

## Abstract

We present a new generalized Dicke model, an impurity-doped Dicke model (IDDM), by the use of an impurity-doped cavity-Bose-Einstein condensate (BEC). It is shown that the impurity atom can induce Dicke quantum phase transition (QPT) from the normal phase to superradiant phase at a critic value of the impurity population. It is found that the impurity-induced Dicke QPT can happen in an arbitrary field-atom coupling regime while the Dicke QPT in the standard Dicke model occurs only in the strong coupling regime of the cavity field and atoms. This opens the possibility to realize the control of quantum properties of a macroscopic-quantum system (BEC) by using a microscopic quantum system (a single impurity atom).

## Introduction

In recent years ultracold atoms in optical cavities have revealed themselves as attractive new systems for studying strongly-interacting quantum many-body theories. Their high degree of tunability makes them especially attractive for this purpose. One example, which has been extensively studied theoretically and experimentally, is the Dicke quantum phase transition (QPT) from the normal phase to the superradiant phase with a Bose-Einstein condensate (BEC) in an optical cavity^1–10^. The Dicke model^11^ describes a large number of two-level atoms interacting with a single cavity field mode, and predicts the existence of the Dicke QPT^10, 12–15^ from the normal phase to the superradiant phase. However, it is very hard to observe the Dicke QPT in the standard Dicke model, since the critical collective atom-field coupling strength needs to be of the same order as the energy separation between the two atomic levels. Fortunately, strong collective atom-field coupling has realized experimentally in a BEC coupling with a ultrahigh-finesse cavity filed^16, 17^. C. Emary and T. Brandes^18^ first indicated that the Dicke model exhibits a zero-temperature QPT from the normal phase to the superradiant phase in the thermodynamic limit. Then, D. Nagy *et al*.^4^ pointed out that the Dicke QPT from the normal to the superradiant phase corresponds to the self-organization of atoms from the homogeneous into a periodically patterned distribution. Soon after this, the Dicke QPT was experimentally observed in the sense of the self-organization of atoms by using the cavity-BEC system^2^. In the Dicke QPT experimental realization^2^, the normal phase corresponds to the BEC being in the ground state associated with vacuum cavity field state while both the BEC and cavity field have collective excitations in the super-radiant phase. A few extended Dicke models^9, 19^ have been proposed to reveal rich phase diagrams and exotic QPTs, which are different from those in the original Dicke model.

Impurities in a BEC have motivated the investigation of a wide range of phenomena^20–33^. For instance, a single impurity can probe superfluidity^20, 21^. A neutral impurity can self- localize in BECs^22–25^, and can be dressed into a quasiparticle, the Bose polaron^26–30^ and the soliton for very large coupling strength between the impurity atom and BEC^31^. Rydberg impurities in the BEC can be used to engineer the phase file of the BEC, and to produce a Yukawa interaction between impurities through phonon^32^. Recently, several groups^34–38^ have experimentally demonstrated the controlled doping of impurity atoms or ions into the BEC. These experimental progress have paved the way for a coherently interacting hybrid system of individually controllable impurities in a BEC system. The realization of various impurities in a BEC presents a new frontier where microscopic atomic physics meets condensed matter and mesoscopic physics.

In this paper, motivated by the recent experimental progress of cavity-BEC and impurity-doped BEC system we propose a generalized Dicke model, an impurity-doped Dicke model (IDDM), by the use of an impurity-doped cavity-BEC. In our model, the impurity atom is treated as a two-level system (a qubit). Physically, there may exist two ways to realize the impurity qubit. The first one is to choose two proper internal states of the impurity atom to denote the qubit. The second one is to use the double-well qubit^39^ which consists of the presence of one impurity atom in the left or right well of the double well, denoted by |0〉 and |1〉, respectively. The impurity-BEC interaction is tunable by an external magnetic field in the vicinity of Feshbach resonances^40, 41^. The cavity-BEC system adopted in our scheme is the same as that in the Dicke QPT experiment^2^. The IDDM can reduce to the original Dicke model when the impurity-BEC interaction is switched off. We discuss how the presence of an impurity atom modifies the results of the original Dicke model. We show that the impurity atom can induce the Dicke QPT from the normal phase to the superradiant phase with the impurity population being the QPT parameter. It is predicted that the impurity-induced Dicke QPT can happen in an arbitrary coupling regime of the cavity field and atoms while the Dicke QPT in the standard Dicke model occurs only in the strong coupling regime of the cavity field and atoms. This opens a possibility to observe the Dicke QPT in the intermediate and even weak coupling regime of the cavity field and atoms.

## Results

### The impurity-doped Dicke model

**Figure 1:**
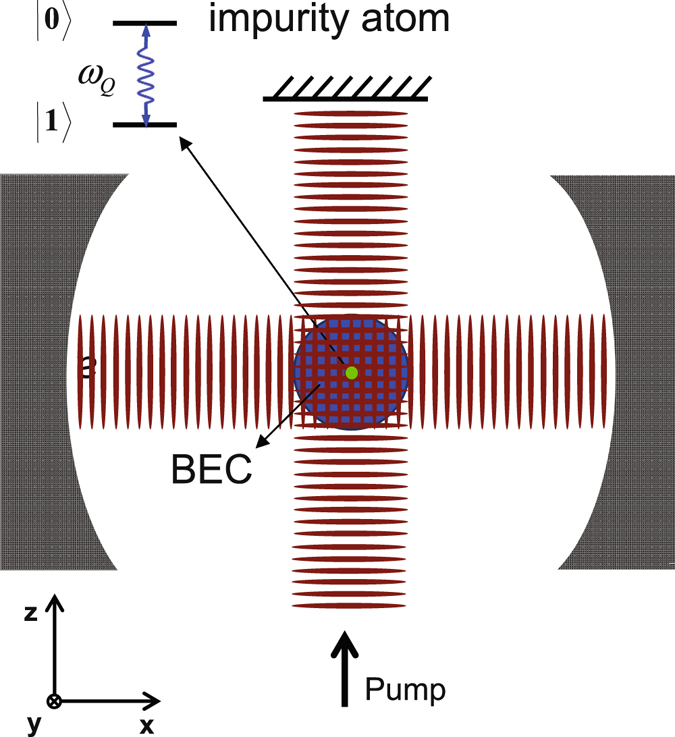
Schematic of the physical system under consideration: An impurity qubit with energy separation *ω*_*Q*_ is doped into a atomic BEC in a ultrahigh-finesse cavity. Both the impurity and BEC couple to a single cavity field and a transverse pump field.

In this section, we establish the IDDM through combining cavity-BEC and impurity-doped BEC techniques. Our proposed experimental setup is indicated in Fig. 1. A two-level impurity atom (qubit) with energy splitting *ω*_*Q*_ is doped in an atomic BEC, which is confined in a ultrahigh-finesse optical cavity.

In the absence of the impurity atom, the cavity-BEC system under our consideration is the same at that employed in the experiments to observe the Dicke QPT^2^. The cavity contains *N* ^87^Rb condensed atoms interacting with a single cavity model of frequency *ω*_*c*_ and a transverse pump field of frequency *ω*_*p*_. The excited atoms may remit photons either along or transverse to the cavity axis. This process couples the zero momentum atomic ground state to the symmetric superposition states of the *k*-momentum states. This yields an effective two-level system. Suppose that the frequency *ω*_*c*_ and *ω*_*p*_ are detuned far from the atomic resonance frequency *ω*_*a*_, the excited atomic state can be adiabatically eliminated. In this case, the single atom Hamiltonian of the system under our consideration can be written as

1 $$\begin{array}{rcl}{\hat{H}}_{\mathrm{(1)}} & = & \frac{{\hat{p}}_{x}^{2}+{\hat{p}}_{z}^{2}}{2m}+(U\,{\cos }^{2}\,k\hat{x}-{{\rm{\Delta }}}_{c}){\hat{a}}^{\dagger }\hat{a}\\  &  & +V\,{\cos }^{2}\,(k\hat{z})+\eta ({\hat{a}}^{\dagger }+\hat{a})\,\cos \,(k\hat{x})\,\cos \,(k\hat{z}).\end{array}$$

Here the first term is the kinetic energy of the atom with momentum operators ${\hat{p}}_{x,z}$. The second term describes the cavity field, where ${\hat{a}}^{\dagger }(\hat{a})$ is the creation (annihilation) operator of the cavity field, which satisfy the bosonic commutation $[\hat{a},{\hat{a}}^{\dagger }]=1$, $U=\frac{{g}_{0}^{2}}{{{\rm{\Delta }}}_{a}}$ is the light shift induced by the atom where *g*_0_ is the atom-cavity coupling strength, Δ_*a*_ = *ω*_*p*_ − *ω*_*a*_ and Δ_*c*_ = *ω*_*p*_ − *ω*_*c*_, *k* is the wave-vector, which is approximated to be equal on the cavity and pump fields. The third term describe the potential along the *z*-axis created by the pump field, the depth of the potential $V={{\rm{\Omega }}}_{p}^{2}/{{\rm{\Delta }}}_{a}$ controlled by the maximum pump Rabi frequency Ω_*p*_. The last term is the potential induced by the scattering between the cavity field and the pump field, where *η* = *g*_0_Ω_*p*_/Δ_*a*_. The atom can be excited from the zero-momentum state |*p*_*x*_, *p*_*z*_〉 = |0, 0〉 to the *k*-momentum state $|{p}_{x},{p}_{z}\rangle ={\sum }_{{\upsilon }_{1},{\upsilon }_{2}=\pm 1}\,|{\upsilon }_{1}k,{\upsilon }_{2}k\rangle$ through the scattering between the cavity field and the pump field due to the conservation of momentum. So the atomic field can be expanded in terms of two-mode approximation $\hat{{\rm{\Psi }}}={{\rm{\Phi }}}_{0}{\hat{h}}_{0}+{{\rm{\Phi }}}_{1}{\hat{h}}_{1}$, where ${\hat{h}}_{0}$ and ${\hat{h}}_{1}$ are bosonic operators and Φ_0_ (Φ_1_) is the zero (k)-momentum single atom wave function. Here $N={\hat{h}}_{0}^{\dagger }{\hat{h}}_{0}+{\hat{h}}_{1}^{\dagger }{\hat{h}}_{1}$ represents the total number of condensed atoms, which holds conservation in this paper. Substituting $\hat{{\rm{\Psi }}}={{\rm{\Phi }}}_{0}{\hat{h}}_{0}+{{\rm{\Phi }}}_{1}{\hat{h}}_{1}$ into the second quantization form

2 $$\begin{array}{rcl}{\hat{H}}_{CB} & = & \int {\hat{{\rm{\Psi }}}}^{\dagger }(x,z){\hat{H}}_{\mathrm{(1)}}\hat{{\rm{\Psi }}}(x,z){\rm{d}}x\,{\rm{d}}z\\  &  & +\frac{s}{2}\int {\hat{{\rm{\Psi }}}}^{\dagger }(x,z){\hat{{\rm{\Psi }}}}^{\dagger }(x,z)\hat{{\rm{\Psi }}}(x,z)\hat{{\rm{\Psi }}}(x,z){\rm{d}}x\,{\rm{d}}z,\end{array}$$

where $s=2\sqrt{2\pi }a/m{l}_{y}$, *a* being s-wave scattering length and *l*_*y*_ being trapped length in the *y* direction. If one introduces the collective spin operators ${\hat{J}}_{z}=({\hat{h}}_{1}^{\dagger }{\hat{h}}_{1}-{\hat{h}}_{0}^{\dagger }{\hat{h}}_{0})/2$, ${\hat{J}}_{+}={{\hat{J}}_{-}}^{\dagger }={\hat{h}}_{1}^{\dagger }{\hat{h}}_{0}$, up to a constant term we obtain a extended Dicke model about the cavity-BEC system

3 $${\hat{H}}_{CB}=\omega {\hat{a}}^{\dagger }\hat{a}+{\omega }_{0}{\hat{J}}_{z}+\frac{\lambda }{\sqrt{N}}(\hat{a}+{\hat{a}}^{\dagger })\,({\hat{J}}_{+}+{\hat{J}}_{-})+\frac{\chi }{N}{\hat{J}}_{z}^{2},$$

where the effective frequency of the cavity field *ω* = −Δ_*c*_ + *NU*_0_/2 and the atomic effective transition frequency *ω*_0_ = *ω*_*r*_ + *χ*′, where *ω*_*r*_ = *k* ^2^/*m* with *k* ^2^/2*m* being recoil frequency and *χ*′ = (*N* − 1) (*χ*_1_ − *χ*_0_)/2 with ${\chi }_{\mathrm{1(0)}}=s\int \,dxdz{|{{\rm{\Phi }}}_{\mathrm{1(0)}}(x,z)|}^{4}$ being the intraspecies coupling strength. $\lambda =\sqrt{N}{g}_{0}{{\rm{\Omega }}}_{p}/2{{\rm{\Delta }}}_{a}$ is the coupling strength induced by the cavity field and pump field, where Ω_*p*_ denotes the maximum pump Rabi frequency which can be adjusted by the pump power. The nonlinear coupling strength is given by *χ* = *N*[(*χ*_0_ + *χ*_1_)/2 − *χ*_01_] with ${\chi }_{01}=s\int \,dxdz{|{{\rm{\Phi }}}_{0}(x,z)|}^{2}{|{{\rm{\Phi }}}_{1}(x,z)|}^{2}$ being interspecies coupling strength.

Next we consider interactions between the impurity qubit and the cavity-BEC. The impurity simultaneously interacts with the BEC, the cavity field, and the pump field. Firstly, we consider the impurity-BEC interaction. We assume that the impurity interacts with the condensates via coherent collisions and only the upper state |0〉 interacts with the condensate considering its state-dependent trapped potential. Similar treatment can also be found in the ref. 42. Neglecting the constant term, the impurity-BEC coupling Hamiltonian has the form

4 $${\hat{H}}_{QB}=-\kappa ({\hat{\sigma }}_{z}+\mathrm{1)}{\hat{J}}_{z},$$

where ${\hat{\sigma }}_{z}$ is the Pauli operator of the impurity qubit and the impurity-BEC coupling strength *κ* = (*κ*_0_ − *κ*_1_)/2, where ${\kappa }_{\mathrm{0(1)}}=2\sqrt{2\pi }b/(M\sqrt{{l}_{y}{l}_{y}^{^{\prime} }})$ $\int \,dxdz{|{{\rm{\Phi }}}_{\mathrm{0(1)}}(x,z)|}^{2}{|{\phi }_{0}(x,z)|}^{2}$ is the coupling strength between the impurity and zero(*k*)- momentum component BEC with *M* being the reduced mass, ${l}_{y}^{^{\prime} }$ being the trapped length of the impurity in *y* direction, *φ*_0_(*x*, *z*) being the wave function of the impurity in the upper state and *b* being the *s*– wave scattering length. In a frame rotating with the pump field frequency *ω*_*p*_, the Hamiltonian of impurity qubit interacting with the cavity field and the pump field reads as

5 $${\hat{H}}_{QF}=-{{\rm{\Delta }}}_{c}{\hat{a}}^{\dagger }\hat{a}+\frac{{{\rm{\Delta }}}_{2}}{2}{\hat{\sigma }}_{z}+{g}_{Q}({\hat{a}}^{\dagger }{\hat{\sigma }}_{-}+\hat{a}{\hat{\sigma }}_{+})+{{\rm{\Omega }}}_{Q}({\hat{\sigma }}_{+}+{\hat{\sigma }}_{-}),$$

where Δ_2_ = *ω*_*Q*_ − *ω*_*p*_ is the detuning between the energy separation of the impurity qubit *ω*_*Q*_ and the pump field frequency *ω*_*p*_, ${\hat{\sigma }}_{+}({\hat{\sigma }}_{-})$ is the raising (lowering) operator of the impurity qubit, *g*_*Q*_ the coupling strength between the impurity qubit and the cavity field, Ω_*Q*_ the pump Rabi frequency. The Hamiltonian ${\hat{H}}_{QF}$ can be divided into two parts:

6 $${\hat{H}}_{QF}={\hat{H}}_{QF}^{\mathrm{(0)}}+{\hat{H}}_{QF}^{\mathrm{(1)}},$$

with ${\hat{H}}_{QF}^{\mathrm{(0)}}=-{{\rm{\Delta }}}_{c}{\hat{a}}^{\dagger }\hat{a}+\frac{{{\rm{\Delta }}}_{2}}{2}{\hat{\sigma }}_{z}$ and ${\hat{H}}_{QF}^{\mathrm{(1)}}={g}_{Q}({\hat{a}}^{\dagger }{\hat{\sigma }}_{-}+\hat{a}{\hat{\sigma }}_{+})+{{\rm{\Omega }}}_{Q}({\hat{\sigma }}_{+}+{\hat{\sigma }}_{-})$. In the far-detuning regime (${g}_{Q}\ll |{{\rm{\Delta }}}_{1}|=|{\omega }_{Q}-{\omega }_{c}|$, ${{\rm{\Omega }}}_{Q}\ll |{{\rm{\Delta }}}_{2}|$), one can introduce a anti-hermitian operator $\hat{S}={g}_{Q}/{{\rm{\Delta }}}_{1}({\hat{a}}^{\dagger }{\hat{\sigma }}_{-}-\hat{a}{\hat{\sigma }}_{+})+$ ${{\rm{\Omega }}}_{Q}/{{\rm{\Delta }}}_{2}({\hat{\sigma }}_{-}-{\hat{\sigma }}_{+})$ to transform the Hamiltonian in Eq. (5) as

7 $$\begin{array}{rcl}{\hat{H}}_{QF}^{^{\prime} } & = & \exp (-\hat{S}){\hat{H}}_{QF}\,\exp (\hat{S})\\  & \approx  & {\hat{H}}_{QF}^{\mathrm{(0)}}+1/2[{\hat{H}}_{QF}^{\mathrm{(1)}},\hat{S}]+{\mathscr{O}}({g}_{Q}^{2}/{{\rm{\Delta }}}_{1}^{2})+{\mathscr{O}}({{\rm{\Omega }}}_{Q}^{2}/{{\rm{\Delta }}}_{2}^{2}).\end{array}$$

Above transformation is called the Fröhlich-Nakajima transformation^43^. Under this transformation, the Hamiltonian in Eq. (5) become the following expression

8 $${\hat{H}}_{QF}^{^{\prime} }=-{{\rm{\Delta }}}_{c}{\hat{a}}^{\dagger }\hat{a}+\frac{{{\rm{\Delta }}}_{Q}}{2}{\hat{\sigma }}_{z}+{\xi }_{1}{\hat{\sigma }}_{z}{\hat{a}}^{\dagger }\hat{a}+{\xi }_{2}{\hat{\sigma }}_{z}({\hat{a}}^{\dagger }+\hat{a}),$$

where ${\xi }_{1}={g}_{Q}^{2}/{{\rm{\Delta }}}_{1}$, ${\xi }_{2}={g}_{Q}{{\rm{\Omega }}}_{Q}/{{\rm{\Delta }}}_{1}+{g}_{Q}{{\rm{\Omega }}}_{Q}/{{\rm{\Delta }}}_{2}$ and ${{\rm{\Delta }}}_{Q}={{\rm{\Delta }}}_{2}+{g}_{Q}^{2}/{{\rm{\Delta }}}_{1}+2{{\rm{\Omega }}}_{Q}^{2}/{{\rm{\Delta }}}_{2}$. Under the Fröhlich-Nakajima transformation, the Hamiltonian ${\hat{H}}_{CB}$ and ${\hat{H}}_{QB}$ will induce impurity-BEC interaction terms ${g}_{Q}{g}_{0}{{\rm{\Omega }}}_{p}/$ $\mathrm{(2}{{\rm{\Delta }}}_{a}{{\rm{\Delta }}}_{1})\,({\hat{\sigma }}_{+}+{\hat{\sigma }}_{-})\,({\hat{J}}_{+}+{\hat{J}}_{-})-\kappa {{\rm{\Omega }}}_{Q}/{{\rm{\Delta }}}_{2}({\hat{\sigma }}_{+}+{\hat{\sigma }}_{-}){\hat{J}}_{z}$ and an impurity-cavity-BEC interaction term $-\kappa {g}_{Q}/{{\rm{\Delta }}}_{1}$ $({\hat{a}}^{\dagger }{\hat{\sigma }}_{-}+\hat{a}{\hat{\sigma }}_{+})\,{\hat{J}}_{z}$. Under the large detuning condition ${g}_{0}{{\rm{\Omega }}}_{p}/2{{\rm{\Delta }}}_{a},\,|\kappa |\ll {g}_{Q},{{\rm{\Omega }}}_{Q}$, these terms can be neglected. Hence, combining Eq. (3) with Eqs (4) and (6) we arrive at the total Hamiltonian of the IDDM

9 $$\begin{array}{rcl}\hat{H} & = & (\omega +{\xi }_{1}{\hat{\sigma }}_{z}){\hat{a}}^{\dagger }\hat{a}+[{\omega }_{0}-\kappa ({\hat{\sigma }}_{z}+\mathrm{1)]}{\hat{J}}_{z}\\  &  & +\frac{\chi }{N}{\hat{J}}_{z}^{2}+\frac{{{\rm{\Delta }}}_{Q}}{2}{\hat{\sigma }}_{z}+\frac{\lambda }{\sqrt{N}}(\hat{a}+{\hat{a}}^{\dagger })\,({\hat{J}}_{+}+{\hat{J}}_{-})\\  &  & +{\xi }_{2}{\hat{\sigma }}_{z}(\hat{a}+{\hat{a}}^{\dagger }).\end{array}$$

The IDDM Hamiltonian reduces to that of the original Dicke model when the impurity-cavity-BEC interactions are switched off (i.e., *κ* = 0,*ξ*_1_ = *ξ*_2_ = 0) and the atomic nonlinear interaction in the BEC vanishes (i.e., *χ* = 0).

#### Dicke quantum phase transition

We now study quantum phases and QPTs in the IDDM proposed in the previous section. Ground-state properties of the IDDM can be analyzed in terms of Holstein-Primakoff transformation^44^ due to the large number of atoms in the BEC. From the Hamiltonian (9), we can see that the properties of the cavity-BEC system is related to the initial state of the impurity qubit. We consider the impurity qubit as a control tool over the cavity-BEC system which is the controlled target system. Let the impurity population *δ* = 〈*σ*_*z*_〉, and make use of Holstein-Primakoff transformation to represent the angular momentum operators as single-mode bosonic operators ($[\hat{c},\,{\hat{c}}^{\dagger }]=1$)

10 $$\begin{array}{l}{\hat{J}}_{+}={\hat{c}}^{\dagger }\sqrt{N-{\hat{c}}^{\dagger }\hat{c}},\quad {\hat{J}}_{-}=\sqrt{N-{\hat{c}}^{\dagger }\hat{c}}\hat{c},\\ {\hat{J}}_{z}={\hat{c}}^{\dagger }\hat{c}-N\mathrm{/2}.\end{array}$$

After taking the mean value over a quantum state of the impurity atom we can rewrite the Hamiltonian (9) as the following form

11 $$\begin{array}{rcl}\hat{H}^{\prime}  & = & {f}_{1}{\hat{a}}^{\dagger }\hat{a}+{f}_{2}{\hat{c}}^{\dagger }\hat{c}+{\xi }_{2}\delta (\hat{a}+{\hat{a}}^{\dagger })+\frac{\chi }{N}{({\hat{c}}^{\dagger }\hat{c})}^{2}\\  &  & +\frac{\lambda }{\sqrt{N}}(\hat{a}+{\hat{a}}^{\dagger })\,({\hat{c}}^{\dagger }\sqrt{N-{\hat{c}}^{\dagger }\hat{c}}+\sqrt{N-{\hat{c}}^{\dagger }\hat{c}}\hat{c}),\end{array}$$

where we have neglected a constant term, and effective frequencies of the two bosonic modes are given by

12 $${f}_{1}=\omega +{\xi }_{1}\delta ,\quad {f}_{2}={\omega }_{r}-\chi ^{\prime\prime} -\kappa \mathrm{(1}+\delta ),$$

which clearly indicate that the impurity atom induces frequency shifts of the cavity mode and the atomic mode. Here the interatomic interacting parameter $\chi ^{\prime\prime} =N({\chi }_{0}-{\chi }_{01})=Ns\int \,dxdz{|{{\rm{\Phi }}}_{0}|}^{2}\,({|{{\rm{\Phi }}}_{0}|}^{2}-{|{{\rm{\Phi }}}_{1}(x,z)|}^{2})$. From the expression of *f*_2_ in Eq. (12) we can see that the presence of the interatomic nonlinear interaction described by the parameter *χ*″ can be understood as the reduction of the recoil energy of the atoms from *ω*_*r*_ to *ω*_*r*_ − *χ*″.

In order to describe the collective behaviors of the condensed atoms and the photon, one can introduce new bosonic operators $\hat{d}=\hat{a}+\sqrt{N}\alpha$ and $\hat{b}=\hat{c}-\sqrt{N}\beta$ ^18^, where *α* and *β* are real numbers. Substituting bosonic operators $\hat{d}$ and $\hat{b}$ into the Hamiltonian (11) and neglecting terms with *N* in the denominator, the Hamiltonian (11) can be expanded by

13 $$\hat{H}^{\prime} =N{E}_{0}+\sqrt{N}{\hat{H}}_{1}+{\hat{H}}_{2},$$

where we ${E}_{0},\,{\hat{H}}_{1}$ and ${\hat{H}}_{2}$ are defined by

14 $${E}_{0}={f}_{1}{\alpha }^{2}+{f}_{2}{\beta }^{2}+\chi {\beta }^{4}-4\lambda K\alpha \beta ,$$

15 $$\begin{array}{rcl}{\hat{H}}_{1} & = & [2\lambda \alpha (K-\frac{{\beta }^{2}}{K})-{f}_{2}\beta -2\chi {\beta }^{3}]\,(\hat{b}+{\hat{b}}^{\dagger })\\  &  & +({f}_{1}\alpha -2\lambda K\beta )\,(\hat{d}+{\hat{d}}^{\dagger })-2{\xi }_{2}\delta \alpha ,\end{array}$$

16 $$\begin{array}{rcl}{\hat{H}}_{2} & = & {f}_{1}{\hat{d}}^{\dagger }\hat{d}+({f}_{2}+2\chi {\beta }^{2}+\frac{2\lambda \alpha \beta }{K})\,{\hat{b}}^{\dagger }\hat{b}\\  &  & +\lambda (K-\frac{{\beta }^{2}}{K})\,(\hat{d}+{\hat{d}}^{\dagger })\,(\hat{b}+{\hat{b}}^{\dagger })-\frac{\lambda \alpha \beta }{K}\\  &  & +[\chi {\beta }^{2}+\frac{\lambda \alpha \beta \mathrm{(2}+{\beta }^{2})}{2{K}^{3}}]\,{(\hat{b}+{\hat{b}}^{\dagger })}^{2}+{\xi }_{2}\delta (\hat{d}+{\hat{d}}^{\dagger }),\end{array}$$

where we have introduced the parameter $K=\sqrt{1-{\beta }^{2}}$. The collective excitation parameters *α* and *β* can be determined from the equilibrium conditions ∂*E*_0_/∂*α* = 0 and ∂*E*_0_/∂*β* = 0, which leads to the following two equations

17 $${f}_{1}\alpha -2\lambda K\beta =\mathrm{0,}\quad 2\lambda \alpha (K-\frac{{\beta }^{2}}{K})-{f}_{2}\beta -2\chi {\beta }^{3}=0,$$

from which we can obtain an equation governing the fundamental features of the QPT in the IDDM

18 $$\beta \,[\mathrm{(2}\chi {f}_{1}+8{\lambda }^{2}){\beta }^{2}+{f}_{1}{f}_{2}-4{\lambda }^{2}]=0.$$

Now we discuss quantum phases and QPT in the impurity-doped Dicke model. For the convenience of discussion, we choose the range of interatomic nonlinear interaction *χ*∈[0,∞). When *f*_1_ *f*_2_ ≥ 4*λ* ^2^, from Eq. (18) we can find *α* = *β* = 0 due to 2*χf*_1_ + 8*λ* ^2^ > 0. This means that both the condensed atoms and the photon have not collective excitations. Hence the cavity-BEC system is in the normal phase. However, when *f*_1_ *f*_2_ < 4*λ* ^2^, from Eqs (17) and (18) we can obtain the two nonzero collective excitation parameters

19 $${\alpha }^{2}=\frac{{\lambda }^{2}\mathrm{(4}{\lambda }^{2}-{f}_{1}{f}_{2})\,\mathrm{(4}{\lambda }^{2}+{f}_{1}{f}_{2}+2\chi {f}_{1})}{{f}_{1}^{2}{(\chi {f}_{1}+4{\lambda }^{2})}^{2}},\quad {\beta }^{2}=\frac{4{\lambda }^{2}-{f}_{1}{f}_{2}}{2\chi {f}_{1}+8{\lambda }^{2}},$$

Eq. (19) implies that there exist macroscopic quantum population of the collective excitations of the condensed atoms and the photon in the IDDM. In this case, the cavity-BEC system is in the superradiant phase. The Dicke QPT is the QPT from the normal phase to the superradiant phase.

From the QPT equation (18) we can see that there exist two independent QPT parameters, the cavity-field-atom coupling strength *λ* and the impurity population parameter *δ*. This is one important difference between the IDDM and the original Dicke model in which there is only one QPT parameter, the coupling strength *λ*. Through the analysis below, we can see that it is the new QPT parameter *δ* that makes the IDDM to reveal new QPT characteristics which do not appear in the original Dicke model. In the following, we investigate the QPT in the IDDM for the three cases: (1) *δ* is the QPT parameter with *λ* being an arbitrary fixed parameter; (2) *λ* is the QPT parameter with *δ* being an arbitrary fixed parameter; (3) Both *λ* and *δ* are independent QPT parameters.

In the first case, the impurity population *δ* is the QPT parameter while the cavity-field-atom coupling strength *λ* is an arbitrary fixed parameter. So we can understand the QPT as the impurity induced QPT. From the QPT equation (18) we can find that the critical parameter *δ*_*c*_ at the QPT point satisfies the following equation

20 $${\delta }_{c}=\frac{-(\omega \kappa +P)\pm \sqrt{16{\lambda }^{2}{\xi }_{1}\kappa +{(\omega \kappa -P)}^{2}}}{2{\xi }_{1}\kappa },$$

where we have introduced the parameter *P* = *ξ*_1_ *f*_2_, which indicates that there does always exist a critical impurity population *δ*_*c*_ for an arbitrary value of the cavity-field-atom coupling strength *λ*. From Eqs (17) and (18), we can find the two quantum phases of the normal phase and the superradiant phase. The normal phase is in the regime of *δ* < *δ*_*c*_ (*δ* > *δ*_*c*_) when *ξ*_1_ < 0 (*ξ*_1_ > 0), and we have *α* ^2^ = *β* ^2^ = 0. In the superradiant-phase regime, we have nonzero collective excitations which are given in Eq. (19).

From the critical-point equation (20), we can see that the impurity-induced Dicke QPT happens even in the weak coupling regime of the cavity field and atoms. This is one of important differences between the IDDM and the original Dicke model in which the Dicke QPT appears only in the strong coupling regime of the cavity field and atoms. It opens a way to observe the Dicke QPT in the intermediate and even weak coupling regime of the cavity field and atoms.

We can determine the type of QPTs which happen in the IDDM through investigating the nonanalyticity of the scaled energy *E*_0_ at the critical point in the thermodynamic limit *N* → ∞. If the *n*th derivative of *E*_0_ shows nonanalytic behavior then it is an *n*th order QPT. In the normal phase, since the scaled energy *E*_0_ = 0, arbitrary order derivative with respect to the QPT parameter *δ* is zero. In the the superradiant phase, we obtain the scaled energy from Eq. (14) after inserting the Eq. (19) into Eq. (14)

21 $${E}_{0}=-\frac{{\mathrm{(4}{\lambda }^{2}-{f}_{1}{f}_{2})}^{2}}{4{f}_{1}\mathrm{(4}{\lambda }^{2}+\chi {f}_{1})},$$

then we have the first derivative and the second derivative with respect to the QPT parameter *δ*, respectively.

22 $$\frac{\partial {E}_{0}}{\partial \delta }=\frac{\mathrm{(4}{\lambda }^{2}-{f}_{1}{f}_{2})Q}{2{f}_{1}^{2}{\mathrm{(4}{\lambda }^{2}+\chi {f}_{1})}^{2}}$$

23 $$\tfrac{{\partial }^{2}{E}_{0}}{\partial {\delta }^{2}}=\tfrac{\mathrm{(4}{\lambda }^{2}-{f}_{1}{f}_{2})\,\mathrm{(4}{\lambda }^{2}{f}_{1}+\chi {f}_{1}^{2})\tfrac{\partial Q}{\partial \delta }-Q\mathrm{(4}{\lambda }^{2}{f}_{1}+\chi {f}_{1}^{2})\,({\xi }_{1}{f}_{2}+\kappa {f}_{1})-Q\mathrm{(4}{\lambda }^{2}-{f}_{1}{f}_{2})\tfrac{\partial \mathrm{(4}{\lambda }^{2}{f}_{1}+\chi {f}_{1}^{2})}{\partial \delta }}{2{f}_{1}^{3}{\mathrm{(4}{\lambda }^{2}+\chi {f}_{1})}^{3}}$$

where we have introduced the parameter $Q=\mathrm{(4}{\lambda }^{2}+\chi {f}_{1})\,\mathrm{(2}{\lambda }^{2}{\xi }_{1}+\kappa {f}_{1}^{2})+2{\lambda }^{2}{\xi }_{1}{f}_{1}(\chi +{f}_{2})$. At the critical point *δ* = *δ*_*c*_, we have the critical equation *f*_1_ *f*_2_ = 4*λ* ^2^. So it is easy to know that the first derivative of the scaled ground-state energy *E*_0_ is continuous while the second derivative ∂^2^ *E*_0_/∂*δ* ^2^ is discontinuous at the quantum critical point *δ* = *δ*_*c*_. Therefore, we can conclude that the QPT induced by the impurity is the second-order QPT.

In the second case, the cavity-field-atom coupling strength *λ* is the QPT parameter while the impurity population *δ* is an arbitrary fixed parameter. So we can understand the QPT as the cavity-field-atom coupling induced QPT. From the QPT equation (18) we can find that the critical parameter *λ*_*c*_ at the QPT point satisfies the following equation

24 $$4{\lambda }_{c}^{2}-{f}_{1}{f}_{2}=\mathrm{0,}$$

which leads to the critical coupling strength

25 $${\lambda }_{c}=\frac{1}{2}\sqrt{(\omega +{\xi }_{1}\delta )\,[{\omega }_{r}-\chi ^{\prime\prime} -\kappa \mathrm{(1}+\delta )]},$$

which indicates that the critical coupling strength *λ*_*c*_ can continuously vary with the impurity population *δ* (−1 ≤ *δ* ≤ 1). This is another important difference between the IDDM and the original Dicke model in which the QPT critical point ${\lambda }_{c}^{s}=\sqrt{\omega {\omega }_{0}}/2$ cannot be adjusted for fixed parameters *ω* and *ω*_0_. The QPT critical point of the original Dicke model can be recovered from Eq. (25) when we take ${\xi }_{1}=\kappa =\chi ^{\prime\prime} =0$.

From equation (25) it is interesting to note that the Dicke QPT in the present model can happen in the weak coupling regime and even in the case of *λ*_*c*_ = 0 through controlling the interatomic nonlinear interaction *χ*″ and the impurity population *δ*. In fact, in the case of the interatomic attractive interaction, the condition of ${\omega }_{r}-\chi ^{\prime\prime} \sim \kappa$ is realizable experimentally. Under this condition we can get *λ*_*c*_ = 0 when *ω*_*r*_ − *χ*″ = *κ* and *δ* = 0 or when *ω*_*r*_ − *χ*″ = 2*κ* and *δ* = 1. A realistic estimation of the present model parameters can be obtained from recent experiments^2, 45–48^. From the experiments in refs 2 and 45, we find the parameters *ω* ~ MHz, *ω*_*r*_ ~ KHz, {*l*_*x*_, *l*_*y*_, *l*_*z*_} ~ {3.2, 16.6, 3.3} *μ*m, and *N* ~ 10^5^. In the present paper, we expect the nonlinear interaction among condensed atoms can reduce the recoil energy of the atoms. This condition can be obeyed for the BEC with attractive interactions between atoms. According to refs 2, 45–48, stable BECs with the negative *s*-wave scattering lengths can be obtained for Rubidium atoms and Potassium atoms. The stability of the BEC with the attractive interactions between atoms is characterized by the stability parameter *C* = *N*|*a*|/*l*_0_ with *l*_0_ being mean harmonic oscillator length^46^. The condensate becomes unstable when *C* > 0.574. Considering the stability of the condensate, we take *C* ~ 0.1^47, 48^, then estimate the parameter $\chi ^{\prime\prime} \sim N\hslash a/(m{l}_{x}{l}_{y}{l}_{z})\sim {\rm{KHz}}$. Therefore we can make *χ*″ approach *ω*_*r*_ by adjusting the scattering length *a*, trapped lengths *l*_*x*_, *l*_*y*_, *l*_*z*_ and the number of the condensed atoms *N*. The impurity-BEC interacting parameters is estimated as $\kappa \sim \hslash b/(M\sqrt{{l}_{x}{l}_{y}{l}_{z}{l}_{x}^{^{\prime} }{l}_{y}^{^{\prime} }{l}_{z}^{^{\prime} }})\sim {10}^{-3}{\omega }_{r}$ with the trapped lengths $\{{l}_{x}^{^{\prime} },\,{l}_{y}^{^{\prime} },\,{l}_{z}^{^{\prime} }\}\sim \{0.1,\,0.1,\,0.1\}\,\mu {\rm{m}}$ and scattering length *b* ~ −1 nm. In the following numerical investigations, we will take *ω*_*r*_ as the unit of the related parameters, and choose *ω* = 400, *χ*″ = 0.99, *κ* = 0.005 and *ξ*_1_ = 0.001.

The third case is a general situation in which two QPT parameters *δ* and *λ* vary independently. In this case, nonzero collective excitations are given by Eq. (19). In the thermodynamic limit *N* → ∞ we can obtain the scaled population inversion of BEC 〈*J*_*z*_〉/*N* and the scaled intracavity intensity *I*/*N* as

26 $$\langle {J}_{z}\rangle /N={\beta }^{2}-1/2,\quad I/N={\alpha }^{2}.$$

**Figure 2:**
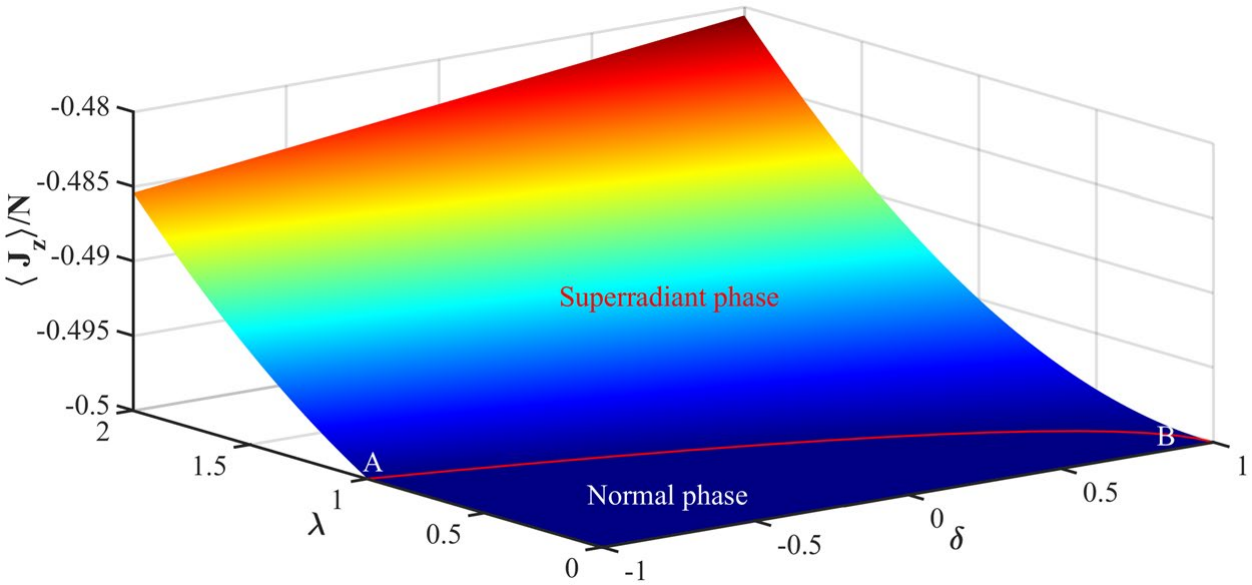
Phase diagrams described by the scaled population inversion of the BEC 〈*J*_*z*_〉/*N* with respect to the impurity population *δ* and the coupling strength *λ*. The related parameters are taken as *ω* = 400, *χ*″ = 0.99, *κ* = 0.005 and *ξ*_1_ = 0.001 in unit of *ω*_*r*_.

We have plotted the phase diagrams of the IDDM for the general case in Fig. 2, which are described by the scaled population inversion of BEC 〈*J*_*z*_〉/*N*. The related parameters are taken as *ω* = 400, *χ*″ = 0.99, *κ* = 0.005 and *ξ*_1_ = 0.001 in unit of *ω*_*r*_. From Fig. 2 we can see that the normal phase is in the region of 〈*J*_*z*_〉/*N* = −0.5 while the superradiant phase is in the region of −0.5 < 〈*J*_*z*_〉/*N* < 0. The Dicke QPT happens at the critical curve *AB* in the phase diagrams indicated in Fig. 2. The critical curve in the phase diagrams appears as the intersection of the two phase regimes for the normal and superradiant phases, and it can be described by the equation

27 $${\lambda }^{2}+\frac{1}{2}\delta -\frac{1}{2}=0.$$

The cavity-BEC is in normal-phase in the regime of ${\lambda }^{2}+\frac{1}{2}\delta -\frac{1}{2} < 0$ and in superradiant phase when ${\lambda }^{2}+\frac{1}{2}\delta -\frac{1}{2} > 0$. In superradiant phase, the collective excitations increase with the QPT parameters *δ* and *λ*.

Finally, we show how to manipulate the impurity population. In order to do this, We introduce an auxiliary atom outside the cavity, which is correlated with the impurity atom. We indicate that the impurity population can be controlled by making projective measurements upon the auxiliary atom. As an example, we consider the case of the impurity atom *A* and the auxiliary atom *B* initially being in the well-known Werner state

28 $$\rho =\frac{1-z}{4}\hat{I}+z|{\rm{\Psi }}\rangle \langle {\rm{\Psi }}|,\quad 0\le z\le \mathrm{1,}$$

where $\hat{I}$ is the unit operator, |Ψ〉 is Bell state $|{\rm{\Psi }}\rangle =({|0\rangle }_{A}{|0\rangle }_{B}+{|1\rangle }_{A}{|1\rangle }_{B})/\sqrt{2}$. In this state, if one dose not measure the auxiliary atom, the impurity population is zero, i.e., $\delta ={{\rm{Tr}}}_{{\rm{AB}}}[\rho {\hat{\sigma }}_{z}^{A})]=0$. We now introduce two orthogonal complete projection operators ${\hat{{\rm{\Pi }}}}_{\pm }^{B}(\theta )={|\psi (\theta )\rangle }_{\pm \pm }\langle \psi (\theta )|$, in which |*ψ*(*θ*)〉_±_ are two orthogonal quantum states of the auxiliary atom with |*ψ*(*θ*)〉_±_ = sin *θ*|1〉 ± cos *θ*|0〉.

For the initial state (28), after making the projective measurements ${\hat{{\rm{\Pi }}}}_{\pm }^{B}(\theta )$ upon the auxiliary atom *B*, we can find that the impurity atom will collapse to the state

29 $${\rho }_{A}^{\pm }=\frac{1-z}{2}\hat{I}+z{|\psi (\theta )\rangle }_{\pm \pm }^{AA}\langle |\psi (\theta )|.$$

From Eq. (29) we can obtain the impurity population *δ*_±_ = ±*z* cos 2*θ*, which indicates that the impurity population depends on the initially state parameter *z* and the angle of the projection measurement *θ* upon the auxiliary atom. Therefore, we can manipulate the impurity population through making projective measurements along different directions upon quantum states of the auxiliary atom.

## Discussion

In conclusion, we have presented a generalized Dicke model, i.e., the IDDM, by the use of an impurity-doped cavity-Bose-Einstein condensate, and investigated QPT properties of the the IDDM. The original Dicke mode can be recovered under certain conditions as a special case of the IDDM. We have shown that the impurity atom can induce the Dicke QPT at a critic value of the impurity population. We have found that the impurity-induced Dicke QPT can happen in an arbitrary coupling regime of the cavity field and condensed atoms while the Dicke QPT in the standard Dicke model occurs only in the strong coupling regime of the cavity field and atoms. Hence, the IDDM reveals new regions of the Dicke QPT. This opens the door to observing the Dicke QPT and studying new physics related to the Dicke QPT in new parameter regimes of the field-atom coupling. It is interesting to note that the impurity atom is a microscopic quantum system while the BEC is a macroscopic quantum system. The impurity-induced Dicke QPT demonstrates that the micro-quantum system can dramatically change quantum properties of the macro-quantum system. On the other hand, if there exists quantum correlations between the external atom and impurity atom in our scheme, no matter how far apart they are, one can control the impurity atom population by manipulating quantum states of the external atom to realize monitoring the Dicke system. This opens the possibility to realize remote control of the macro-quantum system by using micro-quantum system. Based on current experimental developments, we believe that it is possible to observe experimentally the impurity-induced Dicke QPT by measuring the atomic population or the mean photon number of the cavity field.

## Methods

### The derivation of atomic collision interaction Hamiltonian

We first derive the collision interaction Hamiltonian of BEC in Eq. (3). The collision interaction Hamiltonian of BEC is given as

30 $${\hat{H}}_{int}=\frac{s}{2}\int {\hat{{\rm{\Psi }}}}^{\dagger }(x,z){\hat{{\rm{\Psi }}}}^{\dagger }(x,z)\hat{{\rm{\Psi }}}(x,z)\hat{{\rm{\Psi }}}(x,z){\rm{d}}x\,{\rm{d}}z,$$

where $s=2\sqrt{2\pi }a/m{l}_{y}$ with *a* being s-wave scattering length and *l*_*y*_ being trapped length in the *y* direction. Substituting $\hat{{\rm{\Psi }}}(x,z)={{\rm{\Phi }}}_{0}(x,z){\hat{h}}_{0}+{{\rm{\Phi }}}_{1}(x,z){\hat{h}}_{1}$ into above equation, we obtain

31 $${\hat{H}}_{int}={\chi }_{0}{\hat{h}}_{0}^{\dagger 2}{\hat{h}}_{0}^{2}+{\chi }_{1}{\hat{h}}_{1}^{\dagger 2}{\hat{h}}_{1}^{2}+{\chi }_{01}{\hat{h}}_{0}^{\dagger }{\hat{h}}_{0}{\hat{h}}_{1}^{\dagger }{\hat{h}}_{1}$$

where the parameters are derived as

32 $${\chi }_{0}=\frac{s}{2}\int {|{{\rm{\Phi }}}_{0}|}^{4}{\rm{d}}x\,{\rm{d}}z,\quad {\chi }_{1}=\frac{s}{2}\int {|{{\rm{\Phi }}}_{1}|}^{4}{\rm{d}}x\,{\rm{d}}z,\quad {\chi }_{01}=2s\int {|{{\rm{\Phi }}}_{0}|}^{2}{|{{\rm{\Phi }}}_{1}|}^{2}{\rm{d}}x\,{\rm{d}}z$$

Via introducing the collective spin operators ${\hat{J}}_{z}=({\hat{h}}_{1}^{\dagger }{\hat{h}}_{1}-{\hat{h}}_{0}^{\dagger }{\hat{h}}_{0})/2$, $N={\hat{h}}_{1}^{\dagger }{\hat{h}}_{1}+{\hat{h}}_{0}^{\dagger }{\hat{h}}_{0}$, we obtain

33 $${\hat{h}}_{1}^{\dagger }{\hat{h}}_{1}=\frac{N}{2}+{\hat{J}}_{z},\quad {\hat{h}}_{0}^{\dagger }{\hat{h}}_{0}=\frac{N}{2}-{\hat{J}}_{z}.$$

Substituting above equation into Eq. (31), we derive the following Hamiltonian

34 $$=\chi \frac{{\hat{J}}_{z}^{2}}{N}+\chi ^{\prime} {\hat{J}}_{z}+(\frac{{\chi }_{0}+{\chi }_{1}+{\chi }_{01}}{4}){N}^{2}-(\frac{{\chi }_{0}+{\chi }_{1}}{2})N,$$

where the parameters are given as

35 $$\chi =N(\frac{{\chi }_{0}+{\chi }_{1}}{2}-{\chi }_{01}),\quad \chi ^{\prime} =\frac{N-1}{2}({\chi }_{1}-{\chi }_{0})$$

Then we derive the impurity-BEC coupling Hamiltonian in Eq. (4). The impurity-BEC coupling Hamiltonian is written as

36 $${\hat{H}}_{QB}=s^{\prime} |e\rangle \langle e|\int \,{\hat{{\rm{\Psi }}}}^{\dagger }(x,z)\,\hat{{\rm{\Psi }}}(x,z){|{\phi }_{0}(x,z)|}^{2}{\rm{d}}x\,{\rm{d}}z,$$

where $s^{\prime} =2\sqrt{2\pi }b/(M\sqrt{{l}_{y}{l}_{y}^{^{\prime} }})$ with *b* being s-wave scattering length and ${l}_{y}^{^{\prime} }$ being trapped length of the impurity in the *y* direction and *φ*_0_(*x*, *z*) is the wave function of the impurity in the upper state. Substituting $\hat{{\rm{\Psi }}}(x,z)={{\rm{\Phi }}}_{0}(x,z){\hat{h}}_{0}+{{\rm{\Phi }}}_{1}(x,z){\hat{h}}_{1}$ into above equation, we obtain

37 $${\hat{H}}_{QB}\approx {\kappa }_{0}|e\rangle \langle e|{\hat{h}}_{0}^{\dagger }{\hat{h}}_{0}+{\kappa }_{1}|e\rangle \langle e|{\hat{h}}_{1}^{\dagger }{\hat{h}}_{1},$$

where the parameters *κ*_0_ and *κ*_1_ are given as

38 $${\kappa }_{0}=s^{\prime} \int \,{|{{\rm{\Phi }}}_{0}|}^{2}{|\phi |}^{2}{\rm{d}}x\,{\rm{d}}z,\quad {\kappa }_{1}=s^{\prime} \int \,{|{{\rm{\Phi }}}_{1}|}^{2}{|\phi |}^{2}{\rm{d}}x\,{\rm{d}}z$$

Substituting $|e\rangle \langle e|=\mathrm{(1}+{\hat{\sigma }}_{z})/2$ and Eq. (33) into above equation and omitting the constant term, we finally derive the Hamiltonian as

39 $${\hat{H}}_{QB}=-(\frac{{\kappa }_{0}-{\kappa }_{1}}{2})\,({\hat{\sigma }}_{z}+\mathrm{1)}{\hat{J}}_{z}.$$
